# Effects of aerobic interval training on glucose tolerance in children and adolescents with cystic fibrosis: a randomized trial protocol

**DOI:** 10.1186/s13063-019-3803-8

**Published:** 2019-12-26

**Authors:** Karolinne Souza Monteiro, Matheus de Paiva Azevedo, Lucas Menescal Jales, Fernanda Elizabeth Pereira da Silva, Ricardo Fernando Arrais, Karla Morganna Pereira Pinto de Mendonça

**Affiliations:** 1 Department of Physical Therapy, Federal University of Rio Grande do Norte, Natal, Rio Grande do Norte Brazil; 2 Faculty of Health Science of Trairi, Federal University of Rio Grande do Norte, Santa Cruz, Rio Grande do Norte Brasil; 3 Department of Pediatrics, Pediatric and Adolescent Unit of University Hospital Prof. Onofre Lopes of Federal University of Rio Grande do Norte, Natal, Rio Grande do Norte Brazil

**Keywords:** Cystic fibrosis, Glucose tolerance, Aerobic exercise, Exercise, Respiratory function tests

## Abstract

**Background:**

Individuals with cystic fibrosis (CF) may develop CF-related diabetes (CFDR). This comorbidity is related to a poorer quality of life, microvascular complications, a decline in lung function, and an increase in exacerbations, as well as delayed growth and puberty. Evidence exists that physical exercise contributes to glycemic control in individuals with non-CF-related diabetes. This exercise is usually continuous with moderate intensity and long duration, which can cause muscle dyspnea and fatigue in CF individuals. Aerobic interval training (AIT) emerges as a safe and effective alternative for treating these individuals. The objective of this study is to evaluate the effects of AIT on glucose tolerance in children and adolescents with CF.

**Methods:**

This study will be a two-arm, prospectively registered, randomized controlled trial with blind assessors and twenty 6- to 18-year-old individuals with cystic fibrosis (CF) from two different Brazilian states. People with CF will be randomly allocated to either the experimental or control group using block randomization, stratified by puberty stage,. Participants from both groups will receive an educational intervention and will be asked to continue their usual daily treatment for the full duration of the study. Those in the experimental group will perform AIT on a cycle ergometer at home three times a week, for 8 consecutive weeks. The sample characterization will include an assessment of puberty stage, socioeconomic status, dyspnea, and anthropometry. The primary outcome will be the change in glucose tolerance, while the secondary outcomes will include lung function, exercise tolerance, respiratory muscle strength, quality of life, and CF exacerbations. All outcomes will be assessed at baseline, week 9, and week 17.

**Discussion:**

This is the first study to evaluate the effects of AIT on glucose tolerance in children and adolescents with CF. This study will serve as a basis for guiding clinical practice and decision-making in treating glucose intolerance and CF-related diabetes (CFRD) in children and adolescents with CF.

**Trial registration:**

ClinicalTrials.gov Protocol Registration System: NCT03653949. Registered on August 31, 2018.

## Background

Cystic fibrosis (CF) is the most common autosomal recessive, multisystemic, hereditary disease in Caucasians [1]. Due to technological advances, precise knowledge of CF pathophysiology has contributed to the discovery of new treatment approaches. These approaches have increased the life expectancy for individuals with CF [2], which in turn has led to late complications, such as bone disease [3], liver disease [4], depression and anxiety [5], and CF-related diabetes (CFRD) [6].

CFRD is the most common comorbidity in CF [7]. Its prevalence increases with age, ranging from 2% in children to 50% in adults age 30 years or older [6]. The mortality rate is 3.6 times higher in individuals who develop CFRD compared to those who do not [8]. In addition, CFRD is related to a poorer quality of life, microvascular complications, a decline in lung function, an increase in exacerbations, delayed growth, and problems with the development of puberty [9–11].

CFRD is preceded by a long phase of glucose intolerance and periods of postprandial hyperglycemia [12, 13]. It has distinct characteristics that include aspects of type 1 (T1DM) and type 2 diabetes mellitus (T2DM). Its pathophysiology is complex and includes a loss of pancreatic islet cells, which causes insulin and glucagon deficiency, fluctuating insulin resistance, intestinal abnormalities, and liver disease [7, 10].

Physical exercise has been incorporated as a recommended routine for individuals with CF [14]. Evidence exists that exercise decreases the risk of hospitalization [15]; increases the quality of life [16]; increases functional capacity and peak VO_2_ [17]; decreases general and physical fatigue [18]; increases lung function; facilitates airway clearance techniques; improves posture; and, finally, increases muscle strength, flexibility [19], and bone mineral density [19, 20]. The only study known to evaluate the effect of exercise in adults with CFRD reported an improvement in glycemic control [21].

Regarding individuals with glucose abnormalities or non-CF-related diabetes, physical exercise helps in managing blood glucose levels [22–24] and glycated hemoglobin [25]. It also improves the quality of life [22, 26] and increases the VO_2max_ [22] and pulmonary and immune function [24], as well as reducing the risk of cardiovascular disease [22, 24] and mortality [22].

Most physical exercise programs use continuous exercises of moderate intensity and long duration. This intervention increases the treatment time for individuals with CF and can cause unpleasant symptoms such as dyspnea and muscle fatigue [27, 28]. Aerobic interval training (AIT) emerges as a safe and effective alternative for treating individuals with CF [28, 29] because it produces a lower risk of airflow limitation [29], less systemic inflammation [30], less dyspnea, and greater peripheral oxygen saturation [27]. Until now, no studies have evaluated the effect of IT on glycemic control in children and adolescents with CF.

Therefore, this study aims to evaluate the effects of AIT in children and adolescents with CF for the outcomes of glucose tolerance, lung function, exercise tolerance, respiratory muscle strength, quality of life, and CF exacerbations. Our hypothesis is that AIT will improve all the outcomes.

## Methods

### Study design

**Fig. 1:**
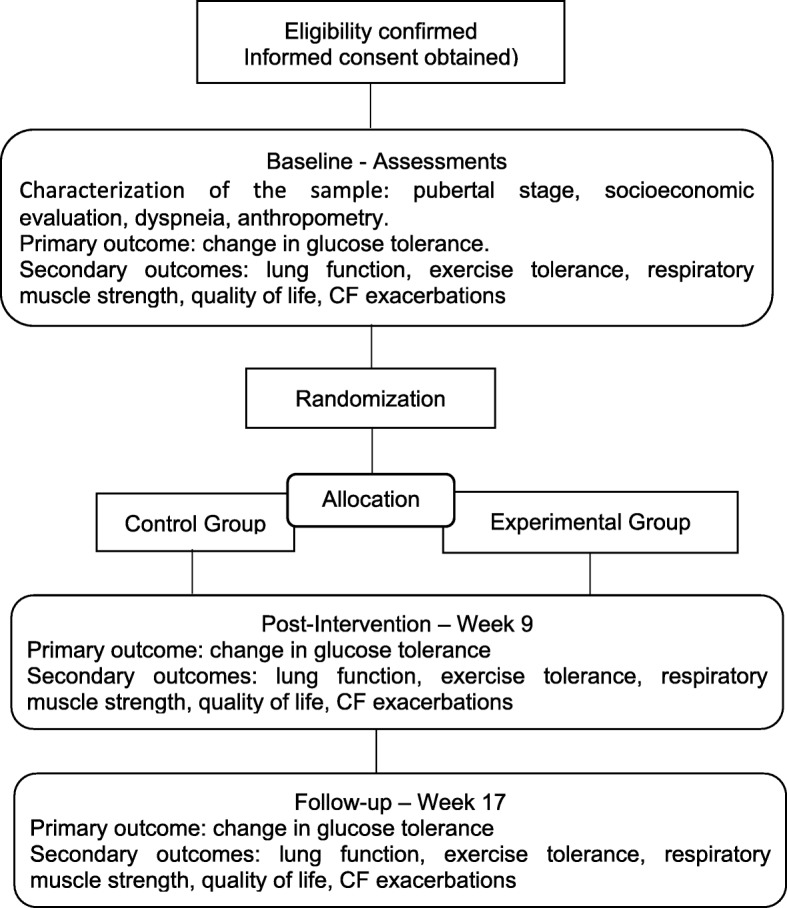
Flow diagram of the planned protocol

This is a two-center, prospectively registered, two-arm, randomized controlled trial with concealed allocation, blinded measurers, intention-to-treat analysis, and 8 weeks of follow-up (Fig. 1). Children and adolescents with CF will be recruited from hospitals specialized in treating the disease in the Brazilian states of Rio Grande do Norte and Paraíba. The recruitment will take place between August 2018 and December 2020.

### Ethical procedures

**Fig. 2:**
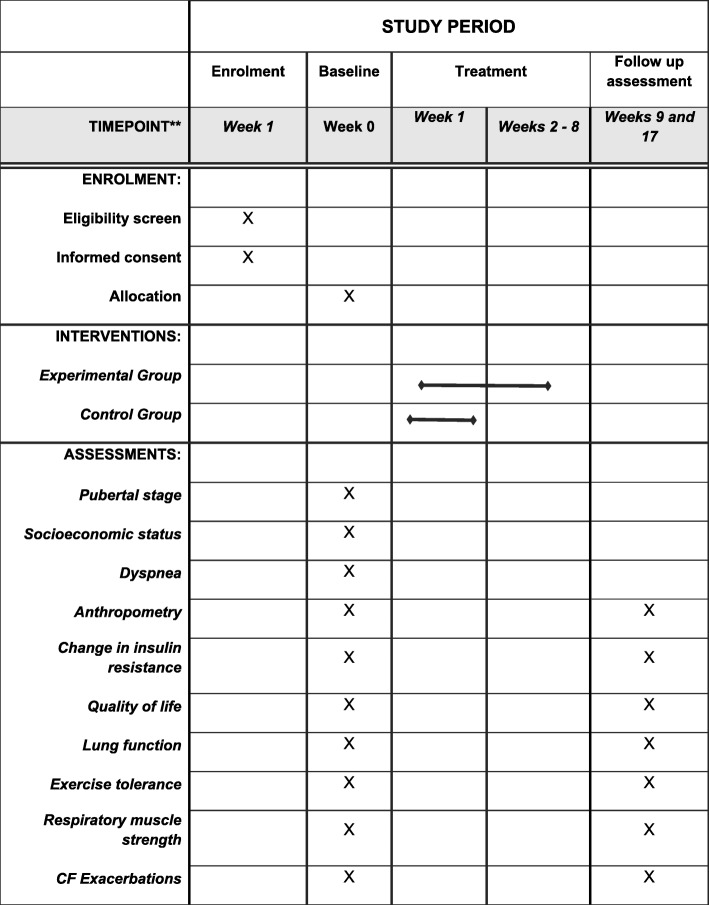
Study design schedule in accordance with the standard protocol items: Recommendations for Interventional Trials (SPIRIT) checklist

The study has obtained ethical approval from the Research Ethical Committee of Universidade Federal do Rio Grande do Norte (CAAE: 88024518.9.1001.5537) under registration number 2.683.882. The trial was prospectively registered at the ClinicalTrials.gov Protocol Registration System (NCT03653949). All participants and their parents will sign an informed consent form prior to participation. The study follows the SPIRIT (Standard Protocol Items: Recommendations for International Trials) 2013 checklist and the TIDieR (Template for Intervention Description and Replication) [31] (Fig. 2 and Additional file 1).

### Inclusion criteria

The inclusion criteria include the following:

- Diagnosis of cystic fibrosis, according to the Brazilian Guidelines for diagnosis and treatment of CF
- Age from 6 to 18 years
- Both sexes
- Not pregnant, in the postpartum period, or lactating.

### Exclusion criteria

The exclusion criteria include the following:

- Present inability to perform the protocol established by the study
- Have exacerbation of the disease, with hospitalization required during the intervention period [32]

### Participants

The population will include children and adolescents aged 6 to 18 years with a medical diagnosis of CF according to the Brazilian Guideline for Diagnosis and Treatment of CF [33]. Children and adolescents with CF will be recruited in a non-probabilistic method in hospitals specialized in treating the disease in the Brazilian states of Rio Grande do Norte and Paraíba.

### Sample size calculation

The sample size calculation was performed by GPower 3.1 software, with a power of 80% and an α of 0.05 being adopted. This procedure was based on the study by Beaudoin et al. (2017) [21], which presents a similar design regarding the same number of groups and the use of exercise as an intervention, although it was developed for adults with CF. In this study, the variable area under the glucose curve after the concurrent exercise program presented values of 34.47 ± 4.23 in the experimental group (EG) and 40.20 ± 11.96 in the control group (CG), with an effect size of 0.30. Based on these parameters, the sample size calculation was 20 participants, with 10 in each group.

The children and the adolescents who discontinue participation in the study will be invited to participate in the assessments 8 weeks after starting treatment, as well as 8 weeks after the end of the intervention. Thus, all individuals will be included in the intention-to-treat analysis.

### Randomization and allocation

Assignment of study participants to the intervention groups will be performed using block randomization [34], with stratification by puberty stage (prepuberty or puberty) [35, 36] being accomplished with the randomization platform found at www.sealedenvelope.com. Participant allocation will be concealed in sequentially numbered and sealed opaque envelopes prepared prior to the study by a research assistant, who will not be involved in the study.

Researcher 1 will be responsible for allocation concealment, researcher 2 will randomize, researcher 3 will conduct an evaluation of the sample while blinded to the groups, researcher 4 will apply the training protocol, and researcher 5 will analyze the data.

### Masking/blinding

Due to the nature of the intervention, the participants cannot be masked. Researcher 3, who will conduct the evaluations, as well as Researcher 5, who will analyze the data, will be blinded to the groups to which the participants are allocated.

### Intervention

The participants from both groups will be instructed to maintain their current nutritional habits, medication, and their regular secretion removal. All participants will be evaluated before the intervention (baseline), in week 9, and in week 17.

### Control group (CG)

The control group and their parents/caregivers will receive an educational intervention, which will be administered through an interactive presentation lasting 20 min. The presentation will address physiopathology, complications, treatments (medical and physiotherapeutic), physical exercises, and prevention of exacerbation. Practical demonstrations of routine care such as the use of inhalation devices, bronchial hygiene techniques, and medication intake will be performed. Participant questions will be answered throughout the presentation [37, 38]. A booklet containing the key points discussed during the presentation will also be distributed (Additional file 2). The intervention will be conducted in week 1, following the baseline assessment.

### Experimental group (EG)

The EG will receive an educational intervention similar to the one conducted with CG, with additional instructions on how to perform the experimental training. The AIT program will be conducted at home, three times a week on alternating days, and using a cycle ergometer for lower limbs (Altmayer Sport). Each session will start with 5 min of warm-up and end with 5 min of cool-down at 30 to 40% of the maximum heart rate (MHR). The training in the initial two weeks will be carried out in six bouts of 20 s, reaching between 70 and 80% of MHR, interspersed by 2 min of active rest, and reaching between 50 and 60% of MHR [33, 39]. Progression will be carried out every 2 weeks by adjusting the time and the amount of the bouts as shown in Table 1.

**Table 1:** Progression of high-intensity interval training protocol for children and adolescents with CF

Week	Warm up	Bout	est	Cool down	Training time	Total time
1	5 min	6 sprints / 20 s	2 min	5 min	14 min	24 min
2	5 min	6 sprints / 20 s	2 min	5 min	14 min	24 min
3	5 min	8 sprints / 20 s	2 min	5 min	18 min 40 s	28 min 40 s
4	5 min	8 sprints / 20 s	2 min	5 min	18 min 40 s	28 min 40 s
5	5 min	8 sprints / 30 s	2 min	5 min	20 min	30 min
6	5 min	8 sprints / 30 s	2 min	5 min	20 min	30 min
7	5 min	10 sprints / 30 s	2 min	5 min	25 min	35 min
8	5 min	10 sprints / 30 s	2 min	5 min	25 min	35 min

Participants will be instructed to hydrate with a quantity of water equivalent to or greater than 500 ml during the training [40]. The intensity of the exercises will be monitored by the frequency meter (Beurer PM15).

Participants will be given a diary before the treatment to record information about the disease exacerbation, heart rate, and the modified Borg scale, as well as to record signs and symptoms observed during training. They will also be shown how to use the diary (Additional file 3). The information will be recorded by the parents and/or caregivers before and after each treatment day. The diary with all the registered information will be returned to the researcher after completion of the treatment for use in evaluating adherence to the proposed intervention. Researcher 4 will maintain weekly contact via cell phone with the parent/guardian to stimulate the intervention and minimize possible deviations from the protocol.

### Sample characterization

A pilot study will initially be carried out with the objective of replicating what will be done during the evaluation and intervention processes, thereby identifying and correcting possible flaws in the proposed procedures. Researcher 3 (who will carry out the evaluations) will undergo training prior to the pilot study.

### Puberty stage

The puberty stage evaluation will be realized by Pediatric Endocrinologist 1 in Natal (Rio Grande do Norte) and Pediatric Endocrinologist 2 in the city of João Pessoa (Paraiba), according to the Tanner scale of puberty stages [35, 36]. The inter-rater reliability will have been previously determined through an evaluation of volunteers performed by both evaluators, and it will be confirmed at a later stage by the kappa statistic. 

### Socioeconomic status evaluation

A questionnaire for sociodemographic and clinical evaluation will be applied that contains information such as gender, date of birth, diagnosis time, number of family members diagnosed with CF, marital status, number of children, and genetic testing. In addition, another questionnaire will be used that is based on the economic classification criteria defined by the Brazilian Association of Research Companies, which categorizes individuals into seven economic classes (A1, B1, B2, C1, C2, D and E). The classification is based on a system of points that considers personal belongings and the individual’s educational level, as well as their access to public services [41].

### Anthropometry

The anthropometric evaluation will consist of measuring height and weight. The subject will be barefoot and wear light clothing for this measurement.

Body weight will be measured in kilograms with a portable scale placed on a regular and firm surface. The individual will stand on an anthropometric mechanical scale (Welmy® Santa Bárbara D’Oeste - SP, Brazil) by homogeneously distributing their weight and maintaining their head in a neutral position. Their weight will then be recorded.

Height will be measured on the same scale. The individual’s legs and feet will be parallel while their arms are relaxed, with the palm of their hands facing their body. Their back will face the wall and their head should settle in a Frankfurt Plane. The stadiometer cursor will be fixed on the individual’s head to record the measurement [42].

### Dyspnea

The modified Medical Research Council (mRC) assesses the degree of dyspnea from zero (without dyspnea, except when in strenuous exercise) to 4 (highly dyspneic when leaving home or dyspneic when dressing up).

### Primary outcome

#### Change in glucose tolerance

The oral glucose tolerance test (OGTT) will be performed to assess the difference in glucose tolerance; for this test, the basal glucose and insulin will be measured in the fasting state. After being tested for this measurement, the individual will ingest 1.75 g/kg of glucose, up to a maximum of 75 g, and the variables will be measured again after 30, 60, 90, and 120 min. Altered tolerance to glucose is diagnosed with values being obtained of between 140 and 199 mg/dL 2 h after glucose consumption. On the other hand, CFRD is diagnosed with glucose values greater than 126 mg/dL in the fasting state or greater than or equal to 200 mg/dL 2 h after glucose consumption. Individuals may present CFRD with fasting (> 126 mg/dL in fasting state) or CFRD without fasting hyperglycemia (< 126 mg/dL in fasting state and > 200 mg/dL 2 h after a glucose overload) [7, 37]. Blood collection will be performed at the University Hospital Onofre Lopes and University Hospital Lauro Wanderley Clinical Analysis Laboratory, using the colorimetric enzymatic method to measure glucose in fluids and the electrochemiluminescence immunoassay for insulin samples.

### Secondary outcomes

#### Lung function

Spirometry will be conducted using a KoKo DigiDoser spirometer (Longmonth, USA), as per the recommendations of the American Thoracic Society (ATS) and the European Respiratory Society (ERS) [43]. Manual calibration of the equipment will be conducted during the examination, following the instructions of the manufacturer and using a 3-L syringe (Vitalograph, Buckingham, England). A disposable mouthpiece and a bactericidal filter (MicroGard 36-MGF1100) will be attached to the apparatus. These parts will be replaced and discarded after each participant use.

A mouthpiece and nasal clip will be used to avoid leaks, and the individual will be instructed on their participation in the examination. The forced vital capacity (FVC), forced expiratory volume in the first second (FEV_1_), Tiffeneau index (FEV_1_/FVC), and forced expiratory flow between 25 and 75% of vital capacity (FEF 25–75) will be recorded in absolute values, as well as in percentages relative to gender, age and height. Up to three acceptable and two reproducible tests can be performed, and the highest value curve is recorded [44]. The values obtained in this study will be compared to the reference values proposed by Mallozi [45].

#### Exercise tolerance

The exercise tolerance of the individuals will be evaluated by the 3-min step test. Participants will be given pre-test counseling and will follow a standardized protocol. They will be instructed to go up and down a 15-cm-high step for 3 min at their normal pace, alternating legs to reduce muscle fatigue. A short 15-s practice will precede the test, and standardized encouragement instructions will be given throughout the test. Their blood pressure will be measured before and after the test. Peripheral oxygen saturation (SpO_2_), heart rate, and modified Borg scale [46] will be assessed before the test; 1, 2, and 3 min into it; and at 1 min after its completion. The total number of times the individual has climbed the step will be recorded at the end of the test. If the participant shows clinical signs of fatigue and/or dyspnea, major changes in vital signs, or report that they are unable to continue, the test should be terminated, and the number of times they climbed the step up to the moment of interruption should be recorded [47–49].

#### Respiratory muscle strength

Respiratory muscle strength will be completed using an analog manovacuometer (GerArd®, São Paulo, SP, Brazil), graduated in cmH_2_O, with an operational interval of ±300 cmH_2_O. This measurement will record the maximum respiratory pressures exerted by the individual. The individual should be seated for this evaluation, with their trunk at a 90° angle with their thighs. They will use a nasal clip to prevent air escape. A demonstration of the examination procedures will be performed before the measurement [50].

Maximal inspiratory pressure (MIP) will be measured using the residual volume and maximum expiratory pressure (MEP) at total lung capacity. Maximal effort can be maintained from 1 to 3 s. The evaluation will be considered complete when the individual performs the maneuver three to five times, with 1-min intervals, to obtain at least two acceptable measures [50, 51]. The reference values will be based on the study by Furtado et al. (2014) [52].

#### Quality of life

The Cystic Fibrosis Questionnaire on Quality of Life, which was translated and validated into Portuguese in 2006, will be used for the quality-of-life evaluation. Four versions of the questionnaire exist, and these are adapted to different age groups: 6 to 11 years old (35 questions), 12 and 13 years old (35 questions), 14 years old or older (50 questions), and parents of children between 6 and 11 years old (44 questions). For children between 6 and 11 years old, the questionnaire will be supplemented with special cards for the child’s responses. The questionnaire addresses the following domains: physical health, body image, digestion, respiration, emotional state, social abilities, nutrition, treatment, vitality, health, social role, and weight. The scores for each domain range from 0 to 100, with a good quality-of-life scoring at or above 50 [53].

#### CF exacerbations

In order to evaluate CF exacerbations, which normally require an increase and/or modification of antibiotics, the following Fuchs criteria adapted by the European Consensus Group will be used [32]: change in volume and/or color of secretion; increases in coughing, fatigue or lethargy; anorexia or weight loss; decrease in lung function of 10% or more as observed on X-ray; and increased dyspnea. The individual must present at least two of these criteria for an exacerbation to be confirmed.

### Patient safety

Adverse events will be monitored in both arms of the trial. Any adverse events that are thought to be causally associated with the intervention will be recorded, managed, and reported to the study coordinators. Serious adverse reactions will be reported to the ethical committee. The study coordinators will compensate participants who experience adverse effects related to the intervention.

### Data management

Data will be collected by researcher 3 directly from the child or adolescent and their parent/guardian. Data will be handled confidentially, and patients will be anonymized. The collected information will be kept for 5 years in accordance with the rules of the Committee of Universidade Federal do Rio Grande do Norte.

All participant data will be recorded in Excel files by researcher 1. All data will be checked by the researcher 1 and rechecked by researcher 2, neither of whom participate in the study’s data collection phase, which ensures the reliability of the data.

### Statistical analysis

Intention-to-treat analysis will be employed to prevent overestimation of intervention efficacy. The numerical data will be presented in averages, while the categorical data will be presented in a frequency distribution. Once data collection is complete, the data distribution normality will be verified by the Shapiro-Wilk test.

Fisher’s Exact Test will be used for intragroup analysis of categorical data, while the two-way mixed-design analysis of variance (ANOVA) test (a repetition factor and a group factor), followed by the Bonferroni post hoc test, will be applied to the numerical data. The effect size (ES) will be analyzed using Cohen’s *f*. If data are non-parametric, the Friedman test with Dunn’s post hoc test will be used for intragroup analysis. The ES will be analyzed using Kendall’s *w*. The Mann-Whitney test will be applied for intergroup analysis, and the ES will be analyzed using Cohen’s *r*. Each ES will interpreted on the basis of Cohen as follows: small (0.21–0.49), medium (0.50–0.79), or large (≥0.80) [54].

All the analyses will consider a confidence interval of 95% (CI95%) and a statistical significance of *P* < 0.05. The Statistical Package for Social Science (SPSS) 20.0 (IBM Corp., Armonk, United States) will be used to analyze the data.

## Discussion

CFRD is preceded by and associated with delayed weight development and loss of lung function, which increase the mortality risks in these individuals [9, 10]. Current treatment uses insulin, but no consensus exists on the treatment of glucose intolerance [33]. Evidence exists that interval exercise promotes reduced plasma glucose [55] and increased insulin sensitivity [56] in individuals with T2DM and in adults with CF [21]. This is the first study to evaluate the effects of AIT on glucose tolerance, function, exercise tolerance, respiratory muscle strength, quality of life, and CF exacerbations in children and adolescents with CF.

This study presents some strengths not present in previous studies, such as the utilization of AIT as an intervention for CF children and adolescents, glucose tolerance outcome, and appropriate assessment instruments. Moreover, this study can be considered of high methodological quality because it is randomized; prospectively recorded; and uses masked evaluators, concealed allocation, and an intention-to-treat approach. If AIT increases the glucose tolerance, it could represent an effective and safe treatment for these individuals. This study will serve as a basis for guiding clinical practice and decision-making in treating glucose intolerance and CFRD in children and adolescents with CF.

A limitation of the study relates to performing the AIT at home without professional supervision. The lack of a professional to supervise the training sessions does not guarantee correct execution of the protocol nor that the training heart rate is reached. However, home exercises more closely reproduce the reality for these children and adolescents, thus enabling identification of reproducible results in any place and promoting the self-management of CF.

## Trial status

Patient recruitment was ongoing at the time of manuscript submission. ClinicalTrials.gov Protocol Registration System: NCT03653949. Registered on August 31, 2018. The study started on February 4, 2019. Data collection will continue until December 2020. URL: https://clinicaltrials.gov/ct2/show/NCT03653949?cond=NCT03653949&rank=1

## Supplementary information

**Additional file 1.** SPIRIT (Standard Protocol Items: Recommendations for Interventional Trials) 2013 Checklist: recommended items to address in a clinical trial protocol and related documents.

**Additional file 2.** A booklet containing the key points discussed during the presentation.

**Additional file 3.** Home data recording diary.

## Data Availability

Datasets analyzed during the current study will be available from the corresponding author upon reasonable request.

## Abbreviations

AIT: aerobic interval training
CF: cystic fibrosis
CG: control group
CFRD: cystic fibrosis-related diabetes
CONSORT: Consolidated Standards of Reporting Trials
EG: experimental group
ES: effect size
FEF 25–75: forced expiratory flow between 25 and 75% of vital capacity
FEV_1_: forced expiratory volume in the first second
FVC: forced vital capacity
MEP: maximum expiratory pressure
MIP: maximal inspiratory pressure
OGTT: oral glucose tolerance test
T1DM: type 1 diabetes mellitus
T2DM: type 2 diabetes mellitus
TIDieR: Template for Intervention Description and Replication
