# Supplementary material for: Effects of aerobic interval training on glucose tolerance in children and adolescents with cystic fibrosis: a randomized trial protocol
Source: Trials. 2019 Dec 26;20:768. doi: 10.1186/s13063-019-3803-8 (PMC6933706; doi:10.1186/s13063-019-3803-8)
Supplement: Supplementary file 2 — Additional file 2. A booklet containing the key points discussed during the presentation. [file 13063_2019_3803_MOESM2_ESM.docx]

Heel Prick test

Sweat test

Gene Mutations test

**Find assistance!**

**In Paraíba:**

Association of Parents and Patients with Cystic Fibrosis of the State of Paraíba – ASPAFIC

**In Rio Grande do Norte:**

Cystic Fibrosis Support

Association of Natal City

Federal University of Rio Grande do Norte Postgraduate Program in Physical Therapy Department of Physical Therapy

How is diagnosis

done?

**Cystic Fibrosis**

##### POWERbreathe ®

**Bronchial hygiene therapy**

Physical Therapy

## References

The Brazilian Cystic Fibrosis Patient Registry. Cystic Fibrosis [Internet]. 2015. p. 1–18. Available from: [http://portalgbefc.org.br/wp- content/uploads/2017/11/Registro2015.pdf](http://portalgbefc.org.br/wp-content/uploads/2017/11/Registro2015.pdf)

How to treat?

Athanazio RA, Silva Filho LVRF da, Vergara AA, Ribeiro AF, Riedi CA, Procianoy E da FA, et al. Brazilian guidelines for the diagnosis and treatment of cystic fibrosis. J Bras Pneumol [Internet]. 2017 Jun;43(3):219–45. Available from: [http://www.scielo.br/scielo.php?script=sci_arttext&pid=S180 6-37132017000300219&lng=en&tlng=en](http://www.scielo.br/scielo.php?script=sci_arttext&amp;pid=S1806-37132017000300219&amp;lng=en&amp;tlng=en)

Google Images:

[https://salusa.pt/4174-large_default/aparelho-para-exerc%C3%ADcio- respirat%C3%B3rio-flutter-powerbreathe-shaker-classic.jpg](https://salusa.pt/4174-large_default/aparelho-para-exerc%C3%ADcio-respirat%C3%B3rio-flutter-powerbreathe-shaker-classic.jpg) [https://http2.mlstatic.com/mascara-nebulizaco-micro-nebulizador-adulto- oxigenio-daru-D_NQ_NP_992682-MLB27008053261_032018-F.jpg](https://http2.mlstatic.com/mascara-nebulizaco-micro-nebulizador-adulto-oxigenio-daru-D_NQ_NP_992682-MLB27008053261_032018-F.jpg)

##### Shaker


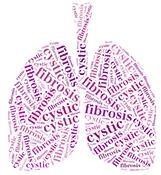

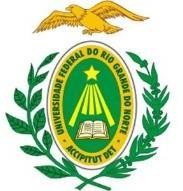

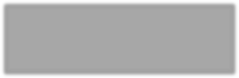

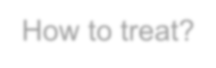

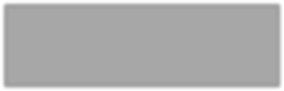

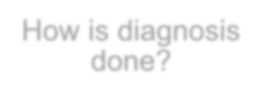

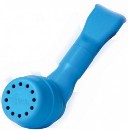

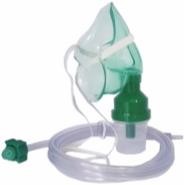

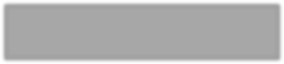

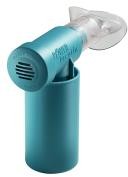


® Nebulization

[https://www.powerbreathe.com/media/catalog/product/cache/1/image/9d f78eab33525d08d6e5fb8d27136e95/c/l/classic-lr_2.jpg](https://www.powerbreathe.com/media/catalog/product/cache/1/image/9df78eab33525d08d6e5fb8d27136e95/c/l/classic-lr_2.jpg)

# What is Cystic Fibrosis?


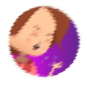

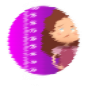

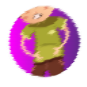

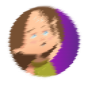

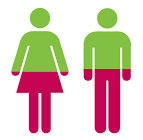

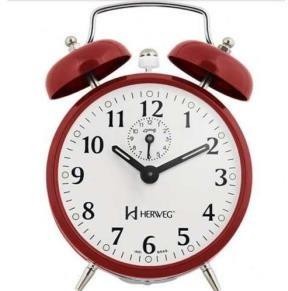

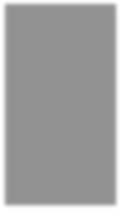

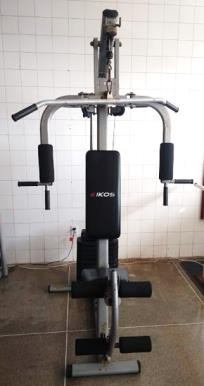

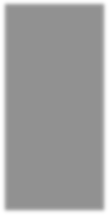

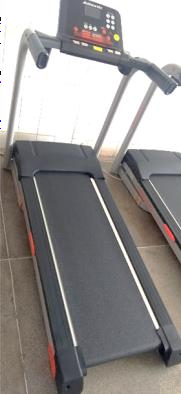

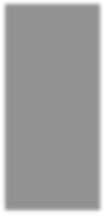

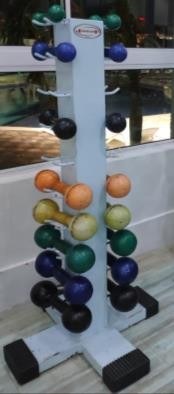

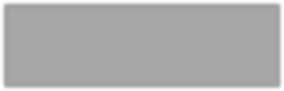

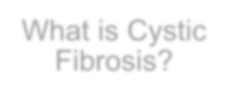

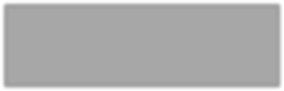

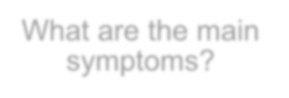

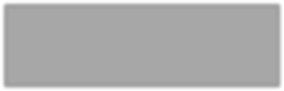

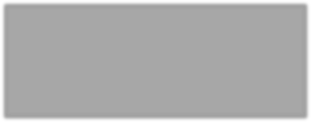

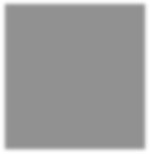

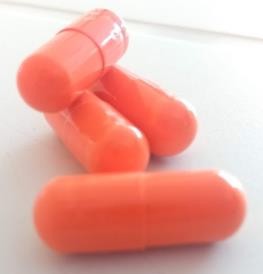

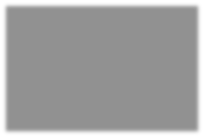

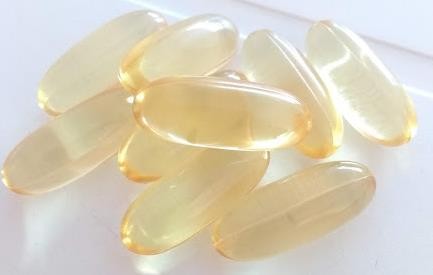


### It’s a genetic disorder that causes lung fluids to become thick and sticky. May affect different systems and patients present different symptoms.

In Brazil, 1 in 7576 newborns are affected

#### 47,2% 52,8%

Medication, vitamins and digestive enzymes

What are the main symptoms?

Chronic cough Developmental disorders Chronic diarrhea

Salty-tasting sweat

**Set aside sufficient time to complete the exercises and data recording!**

#### Plan ahead of time according

Physical Exercise

to your daily activities.

Set up a daily reminder with all the medications.

Try multitasking! Enjoy the nebulization time to study or watch movies.

Make that a habit!

ADHERENCE is the main factor for the success of the treatment
