# Supplementary material for: Effects of aerobic interval training on glucose tolerance in children and adolescents with cystic fibrosis: a randomized trial protocol
Source: Trials. 2019 Dec 26;20:768. doi: 10.1186/s13063-019-3803-8 (PMC6933706; doi:10.1186/s13063-019-3803-8)
Supplement: Supplementary file 3 — Additional file 3. Home data recording diary. [file 13063_2019_3803_MOESM3_ESM.docx]

**Additional file 3 - HOME DATA-RECORDING DIARY**

This is the diary which you will use to record data during your child’s exercises. You must fill-in all fields, on every training day.

If your child experiences a change in the volume and/or color of secretion, increase in coughing, increase in discomfort, fatigue or lethargy, anorexia or weight loss, a decrease of 10% or more in lung function/changes observed under X-rays, increased dyspnea, or use/change of antibiotics, you should report the change in the "exacerbation of condition" line.

| **Week X – from ___/___/___ to ___/___/___** | | | |
| --- | --- | --- | --- |
| **Variables** | **Day 1** | **Day 2** | **Day 3** |
| Heart rate before exercise |  |  |  |
| Borg scale before exercise |  |  |  |
| SpO_2_ before exercise |  |  |  |
| Signs and symptoms during the exercise |  |  |  |
| Heart rate at the end of exercise |  |  |  |
| Borg scale at the end of exercise |  |  |  |
| SpO_2_ at the end of exercise |  |  |  |
| Exacerbation of conditon |  |  |  |
